# The interactions between botulinum-toxin-based facial treatments and embodied emotions

**DOI:** 10.1038/s41598-018-33119-1

**Published:** 2018-10-03

**Authors:** Michael B. Lewis

**Affiliations:** School of Psychology, Cardiff University, Cardiff, CF10 3AT UK

## Abstract

Botulinum toxin (BTX) injections reduce muscle mobility and are commonly used to treat the appearance of glabellar frown lines. Research shows that this cosmetic treatment leads to a reduction in depression. This reduction is consistent with the theory of embodied emotions because patients have a reduced ability to frown and so receive less negative feedback associated with this action. The current research explored this effect and three further hypotheses for the effects of cosmetic BTX injections based on embodied emotions. It was hypothesised that treatment of crow’s feet (or laughter lines) would reduce mood as patients’ Duchenne smiles would be impaired. It was hypothesised that facial BTX treatments would impair emotional expression recognition because the ability to mimic emotions would be reduced. Finally, it was hypothesised that, as BTX treatments prevent facial expressions associated with sexual excitement, sexual function would be impaired after treatment. Twenty four BTX-treated and twelve matched participants (all female) were tested before and after treatment. Results found that BTX treatment of laughter lines was associated with increased depression scores. Further, BTX treatment was associated with reduced emotion recognition ability and sexual function. The current results add to our knowledge of the psychological effects of injections of powerful neurotoxins and broaden the scope of the embodiment of emotions.

## Introduction

The reduction of facial wrinkles using injections of botulinum toxin (BTX) has become a staple of the aesthetic treatment industry. It is the most popular cosmetic procedure with 4.5 million treatments during 2016 in the USA^1^. The pharmacological action of BTX is to reduce the mobility of the targeted facial muscles, which consequentially reduces the appearance of facial lines^2^. There is increasing evidence, however, that these treatments affect the patients’ psychological responses as well as just their muscular actions and appearance of lines. Here, the effects of cosmetic BTX treatments on these psychological responses are considered together with explanations for these findings based on the concept of embodied-emotions. Further hypotheses, in three areas of interest, are derived from the theory of embodied emotions and these are tested in a group of BTX-treated patients and comparison patients. The first area of interest is the effect of BTX treatments on the muscles used to express positive and negative emotions and how these may have negative and positive effects on mood respectively. The second area of interest is how BTX treatments may prevent facial mimicry and whether this might impair emotion recognition. The final area of interest considers the role that facial expressions play during sexual excitement and how BTX treatments might reduce these expressions and hence potentially sexual satisfaction.

The most studied psychological effect of BTX treatments has been the change of mood that results from the treatment of frown lines. It was originally observed that patients treated for glabellar lines with BTX appeared to be happier^3^. This observation could have been in appearance only as these patients had a reduced ability to frown even if they were unhappy; however, it has since been shown that this effect on mood goes beyond just the appearance. Finzi & Wasserman^4^ demonstrated that the treatment of frown lines reduced significantly the symptoms of patients with depression and Lewis & Bowler^5^ demonstrated that there is a general improvement in mood following BTX treatment for frown lines compared to a comparison group who had received other cosmetic treatments. Since these studies, there have been a number of randomised control trials (RCTs) demonstrating the robust effect that BTX treatment of frown lines has on depression^6–11^.

The common explanation for the effect that BTX has on mood derives from the facial feedback hypothesis^12^. This theory suggests that forming a facial expression strengthens the internal feeling of that expression. Demonstrations of this effect include that smiling causes a cartoon to seem funnier^13^. Although there has been a challenge to this original finding^14^, there have been many replications of the principle and a meta-analysis shows a robust facial-feedback effect^15^. A range of emotions have also been shown to be affected by feedback from facial expressions: wrinkling one’s nose makes one more disgusted^16^ and frowning leads to pictures being more negatively evaluated^17^. Emotional feedback is not restricted to facial actions and explorations of the effects that moving one’s body, as well as one’s face, has on emotions has led to the idea of embodied emotion^18,19^. Embodied emotion provides a possible explanation for the effect BTX treatments have on mood: patients who have received treatment for corrugator and procerus muscles (muscle groups targeted in frown line treatments) would be unable to frown and so would not receive the negative affective feedback that is the embodied emotion associated with frowning. The explanation is that the lack of feedback of negative affect leads to the positive effect that BTX treatments have been shown to have on mood.

Embodied emotions and the facial feedback hypothesis were developed under the prevailing view of the basic emotion theory that suggests that emotional expressions are the outward signals of inner feelings^20^. However, more recent work suggests that facial expressions are tools for social influence and the links between emotions and facial expressions may not be as direct as previously thought^21,22^. The alternative behavioral ecology view of facial displays suggests that frowning may not always indicate sadness or anger but might instead be used to recruit protection or elicit submission. On this view, emotions do not always feedforward to facial displays. Embodied emotions and facial feedback can be seen as being potentially consistent with both views of emotional expressions because regardless of whether a frown is originated from a feeling of sadness or an attempt to recruit protection, the theory of embodied emotion predicts that frowning increases sadness. So while there may or may not be feedforward of emotions to expression, there is evidence, as stated above, that expressions feedback to emotions.

It could be argued that an alternative explanation for the BTX effect on mood based on social interactions. The BTX treatment prevents frowning and so the person is less likely to frown at the people around them. Due to the social mimicry of emotional expressions, this means that fewer people will frown back at the person, which could have an overall positive effect on mood. While this alternative explanation is possible, evidence against it comes from the demonstration that BTX treatments affect the activation of the amygdala during a facial mimicry exercise^23^ and so even outside of a social context, BTX is affecting the processing of emotions.

Until now, cosmetic BTX treatments have been shown to have largely positive effects on a person’s mood. Here, the possibility is explored that these treatments may also have negative effects as directly predicted from the ideas of embodied emotions.

## Laughter Lines

Frown lines are not the only facial lines that are commonly treated using cosmetic BTX injections. Lines radiating from the corners of the eyes are often targeted through injections into the orbicularis oculi muscles^24^. These lines are known as crow’s feet or laughter lines and research reported here explores whether pharmacological paralysis of the muscles producing these laughter lines has any effect on mood.

The orbicularis oculi muscles play an important role in smiling as demonstrated by research into the Duchenne smile^25^. A Duchenne smile (or true smile) is one that involves both the mouth and the eyes: the zygomaticus major and the orbicularis oculi muscles are contracted. This can be contrasted with a false smile, which only employs the zygomaticus major and is sometimes referred to as a Pan Am smile or a Botox smile^26^. Research shows that a smile expression using the eye muscles is seen as being warmer and more sincere than one that uses only the mouth muscles^27^. Soussigan^28^ demonstrated that the facial feedback effect from smiling is stronger when the orbicularis oculi muscles are contracted. The potential importance of Duchenne smiles have also been shown in two studies of photographs in yearbooks. These studies showed that people who showed a Duchenne smile in their yearbook divorced less^29^ and lived longer^30^ than those that only smiled only with their mouths. Exactly why these relationships exist is unclear but these studies show that people who tend to smile with their eyes have more positive life outcomes.

Given the importance of the orbicularis oculi muscles in smiling, it would be expected that reducing their mobility by using BTX injections will have psychological consequences. The theory of embodied emotion predicts that reducing a person’s ability to make a Duchenne smile would reduce the facial feedback they receive from a smile. A possible consequence is that a person’s mood will be lower if they have received a BTX treatment that reduces the mobility of their orbicularis oculi muscles. The BTX treatment of crow’s feet does exactly that.

One aim of the current study was to explore the effect of BTX treatment of crow’s feet on mood scores before and after treatment. Participants were recruited that either received BTX for just their frown lines or received BTX for both frown lines and crow’s feet – it is unusual to find people who have had crow’s feet treated but not frown lines. The hypothesis was that those people who had received BTX treatment for crow’s feet would score higher on a measure of depression after treatment than those who received a different treatment.

## Emotion Recognition

Embodied emotion has been shown to affect the recognition of emotional expressions in others and this is explained through the effect of mimicry. Evidence for this is that the recognition of the emotions in other people is improved with facial mimicry^31^. Indeed, preventing mimicry of emotional expressions leads to poorer emotion recognition^32^, whereas encouraging expression mimicry can increase emotion recognition accuracy^33^. It would be expected, therefore, that treatments that reduce the ability to mimic facial expressions of others would lead to reduced accuracy in determining the emotions portrayed in the facial expressions of others.

Research already exists that explores the effects of BTX injections on emotion processing. For example, the time it takes to read an emotional text is lengthened following facial BTX injections^34^. Also, the size of the emotional response to video clips is reduced for BTX treated participants compared with controls^35^. This indicates a reduction in felt emotion, which is confirmed by lower activation in the amygdala when BTX-treated people attempt to mimic an angry expression^23^. Baumeister, Papa & Foroni^36^ tested people who had been treated with BTX for frown lines and matched controls. They found that, compared to controls, BTX treated participants rated slightly emotional sentences as being less emotional. Similarly they found that happy and sad faces were rated as less happy and less sad respectively by the BTX treated group. Finally, happy and sad faces were categorised as such more slowly after the BTX treatment. These studies, together, suggest that there is reduced emotional responding following BTX treatment.

This previous research demonstrates that the strength of an emotional evaluation is reduced or slowed following BTX treatments. What is potentially more interesting is whether there is also a reduction in the accuracy with which emotional expressions are recognised. BTX injections for cosmetics reasons lead to a reduction in the ability to mimic certain facial expressions. Therefore, as mimicry is important for emotional expression recognition, one would expect to find that people who have had cosmetic BTX treatments to be poorer at emotion recognition tasks. Previous research has found that BTX treatment does reduce the ability to determine emotions when expressed in just the eyes^37^ using The Reading the Mind in the Eyes task (RMET)^38^.

The current research builds on this previous research in two ways. First, participants were tested before and after treatments using a prospective design to isolate individual differences. Second, a full-face emotion-recognition task was included, as well as the RMET, in order to assess more natural viewing. This second task was the facial expression recognition (FER) test^39^ which consists of the Ekman faces^40^ blended with neutral faces to vary the difficulty of the task, which is to judge the emotion being shown on the face. It was hypothesized that those who received BTX treatments would show a deficit in emotion recognition tasks following treatment and further this detriment would be greater than that observed in a comparison group who received a cosmetic treatment that did not involve BTX.

## Sexual Pleasure

The current research also explored whether embodied emotion or facial feedback have a role to play in sexual intercourse and successful orgasm. Orgasmic dysfunction has a prevalence rate of between 16–25% for women^41^ and is related to sexual satisfaction. Indeed, the female orgasm has received considerable research interest since the late twentieth century^42^. The role of facial expressions during intercourse is considered here and whether treatments that reduce facial expressions of emotion can impact upon the quality or ease of achieving orgasm.

Only a few studies have looked at facial expressions during sexual intercourse but what research there is suggests that there are consistent involuntary facial actions associated with orgasm. Famously, Master and Johnson^43^ studied behaviour during 10,000 sexual interactions. One of their many observations was the common occurrence of frowns seen during the plateau phase (that is the sexual excitement prior to orgasm). The presence of frowning during sexual excitement was further confirmed by researchers^44^ who coded video clips of people’s faces during masturbation using the Facial Action Coding system^45^. The largest differences in expressions between the plateau stage and either the initial baseline or the resolution stages were for the presence of jaw drop (AU26) and frowning (AU4). Popular culture has coined the term the ‘O face’ to refer to this expression^46^.

Studies of non-human primates have also found consistent patterns of facial expressions during sexual excitement. Stump-tailed macaque demonstrate contraction of the corrugator (or frown) muscles during sexual activity^47,48^. While this finding is suggestive of there being a potential universality to this facial expression, recent research has found some slight cultural variations in the facial expressions produced during sexual pleasure^49^.

There are several reasons for the facial expressions associated with orgasm. First, it could be that the facial expressions improve coital communication providing feedback to the sexual partner. This is consistent with the behavioural ecology view of emotional expression^22^. This communication may act as a reward to the partner or just improve timing and cooperation – either way; this would act to enhance pair bonding. Alternatively, applying embodied emotion to the experience of sexual excitement suggests that the act of frowning is not just a response to sexual excitement but may also support and enhance it. That is, the O face is not just a result of sexual excitement, but it is part of the experience of sexual excitement without which the experience will be reduced. A consequence of this latter explanation is that inhibiting the facial expression of arousal, either through volition or pharmacologically, would reduce the experience of that arousal. The hypothesis tested here, therefore, is that participants’ reports of sexual pleasure will be reduced following BTX injections that prevent the frowning that is associated with sexual excitement.

In order to test the effect of BTX cosmetic treatment on sexual pleasure, the quality of participants’ sexual experience was measured using the Female Sexual Function Index (FSFI)^50^. This measure consists of 19 questions that relate to the six domains of sexual function: desire, arousal, lubrication, orgasm, satisfaction and pain. While the hypothesis suggests that BTX treatment will have a detrimental effect on sexual function as a whole, it was also predicted that the domain of orgasm would be affected most by BTX treatments.

## Experiment

The current experiment compared participants who received BTX cosmetic treatment with those who had received cosmetic treatments that do not affect facial mobility. As people self-selected their treatment, this would be better called a quasi-experiment rather than an experiment. Comparisons were also made between participants who received BTX treatment for frown lines only and those that combined that treatment with BTX treatment for crow’s feet and so there were three groups of participants. The design was naturalistic with participants being recruited who were undergoing self-selected cosmetic treatment but participants were tested prior to the cosmetic treatment and following their treatment to compare each participant with their own baseline measures. Although there were no gender-based selection criteria, all participants were female. The quasi-experiment explored the participants’ mood, their ability to recognise emotional expressions accurately and it included a measure of sexual function.

## Results

### Participant Categorisation

Twenty four of the thirty six participants (all female) reported receiving BTX injections between the first testing session and the second testing session. These injections were to the corrugator and procerus muscles to treat glabellar frown lines. Of these participants, 11 also had further injections into their orbicularis oculi muscles to treat crow’s feet. The comparison group were 12 female participants who had no BTX injections but did receive facial peels, dermal fillers, laser treatment or dermal needling between the first session and the second session. Table 1 provides demographic details of the three groups of participants and details of the timing of the two sessions. The ages of the participants were not significantly different between the three groups, *F*(2, 33) = 1.804, *p* = 0.180. All participants were asked to complete all parts of the procedure on two occasions. Two participants (both in the BTX frown line only condition) choose not to complete the FSFI questionnaire in either session.

**Table 1:** Demographic details for the 36 participants.

	Comparison Group. Non-BTX	BTX frown lines only	BTX frown lines and crow’s feet
N	12	13	11
Average Age	38.8	43.9	45.6
(Range)	28–54	32–65	31–55
**Ethnicity:**			
White	11	13	11
Mixed	1		
Average time between first session and treatment.	8.1 days	9.5 days	6.3 days
Average time between treatment and second session.	36.8 days	40.2 days	41.8 days

These are split according to what BTX treatments they received.

### Mood Questionnaire

**Figure 1:**
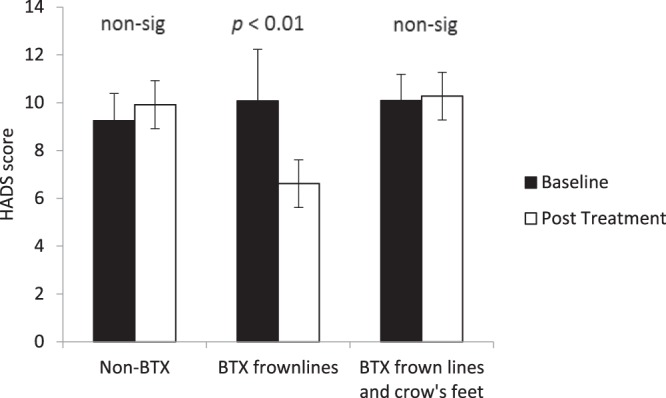
Baseline and post treatment average scores on the HADS mood questionnaire. Participants are categorised according to what type of BTX treatment they received. Error bars show 95% confidence intervals. Significance values show the simple comparisons before and after treatment.

The scores on the Hospital Anxiety and Depression Scale (HADS)^51^ questionnaire show that there was a decrease in negative mood following treatment for the participants who received the BTX treatment for frown lines only whereas a similar decrease was not seen for the non-BTX group nor the BTX frown lines and crow’s feet group (Fig. 1). A two-way ANOVA with factors of treatment type and session found a significant interaction, *F*(2, 33) = 8.564, *p* = 0.001, η^2^ = 0.318. Using planned comparisons, it was revealed that there was a significant improvement in mood (lower HADS scores) for participants who had received BTX treatments for frown lines only, *t*(12) = 3.285, *p* = 0.007, *d* = 1.897, whereas the HADS score were higher, albeit non-significantly, for the participants who had received BTX treatment for crow’s feet as well, *t*(10) = 0.339, *p* = 0.742, *d* = 0.214, and for those who were in the non-BTX group, *t*(11) = 1.201, *p* = 0.255, *d* = 0.724.

The overall HADS score could be split according to a depression element and an anxiety element. A three-way ANOVA including the measure as a factor (depression versus anxiety) found a non-significant treatment-by-session-by-measure interaction, *F*(2, 33) = 0.017, *p* = 0.986, η^2^ = 0.001. Alternatively two separate analyses of the two measures found significant interactions between session and treatment type for both anxiety, *F*(2, 33) = 5.393, *p* = 0.009, η^2^ = 0.246, and depression, *F*(2, 33) = 4.980, *p* = 0.013, η^2^ = 0.232.

### Reading the Mind in the Eyes test

**Figure 2:**
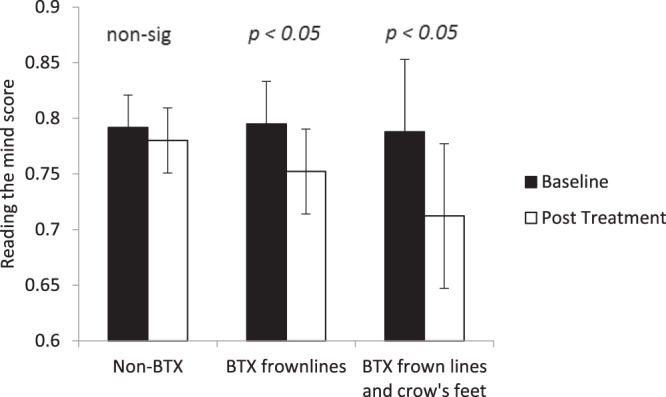
Baseline and post treatment average performance on the Reading the Mind in the Eyes test. Participants are categorised according to what type of BTX treatment they received. Error bars show 95% confidence intervals. Significance values show the simple comparisons before and after treatment.

The responses to the test of emotion recognition based on eye regions only for the 36 images were converted into a single measure of accuracy in terms of proportion correct. Figure 2 shows the overall performance on this test before and after treatment for the two BTX treated groups and non-BTX treated groups. A two-way ANOVA with factors of treatment type and session found an interaction that approached significance, *F*(2, 33) = 3.78, *p* = 0.050, η^2^ = 0.116. This interaction was significant, however, if the two BTX conditions were contrasted against the non-BTX condition, *F*(1, 34) = 4.578, *p* = 0.040, η^2^ = 0.095. Using planned comparisons, it was revealed that there was a significant drop in performance following BTX frown line treatment, *t*(12) = 2.277, *p* = 0.042, *d* = 1.314, and following BTX frown line and crow’s feet treatment, *t*(10) = 2.399, *p* = 0.037, *d* = 1.517, but not following non-BTX treatments, *t*(11) = 0.810, *p* = 0.435, *d* = 0.488.

### Facial Emotion Recognition test

**Figure 3:**
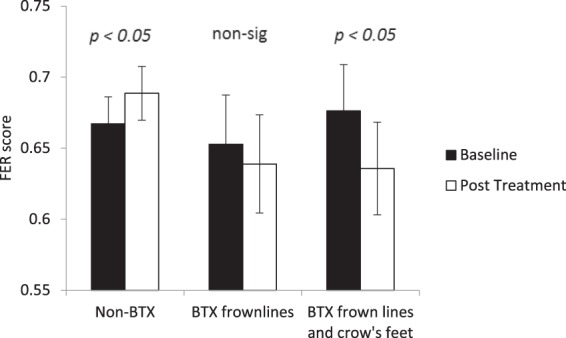
Baseline and post treatment average performance on the Facial Expression Recognition (FER) test. Participants are categorised according to what type of BTX treatment they received. Error bars show 95% confidence intervals. Significance values show the simple comparisons before and after treatment.

The FER test was analysed to obtain a measure of overall accuracy of emotion identification regardless of the strength of the emotion being expressed. Figure 3 shows the performance on this test for the participants before and after treatment. The accuracy measure increased after treatment for non-BTX treated participants but decreased for BTX-treated participants. A two-way ANOVA with factors of treatment type and session found a significant interaction, *F*(2, 33) = 4.375, *p* = 0.021, η^2^ = 0.210. Using planned comparisons, it was revealed that there was was a significant rise in performance for non-BTX treated participants, *t*(11) = 2.321, *p* = 0.040, *d* = 1.400, whereas the fall in performance was not significant for the BTX frown line treated participants, *t*(12) = 0.820, *p* = 0.420, *d* = 0.473, but was significant the BTX frown line and crow’s feet treated participants, *t*(10) = 2.543, *p* = 0.029, *d* = 1.608.

### Female Sexual Function Index

**Figure 4:**
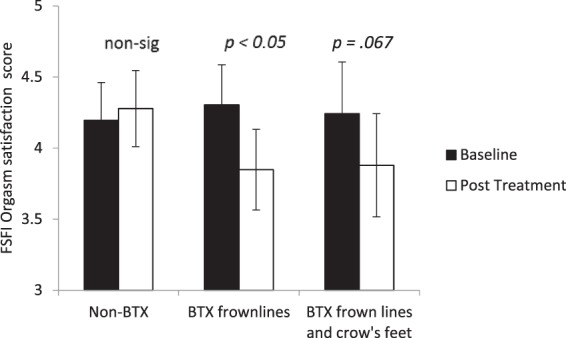
Baseline and post treatment scores on the orgasm subscales of the Female Sexual Function Index (FSFI) categorised according to what type of BTX treatment they received. Error bars show 95% confidence intervals. Significance values show the simple comparisons before and after treatment.

Figure 4 shows the pre-treatment and post-treatment scores on the orgasm component of the FSFI for the two BTX-treated groups and non-BTX-treated group. The BTX-treated groups show a drop in the overall orgasm satisfaction score after treatment regardless of whether the crow’s feet were treated or not – a drop that was not apparent in the non-BTX group. An ANOVA was carried out with factors of session (baseline or post-treatment) and treatment type on the orgasm satisfaction score. The interaction between treatment type and session was significant with scores dropping more following BTX treatments than non-BTX treatments, *F*(2, 31) = 3.650, *p* = 0.038, η^2^ = 0.191. Using planned comparisons, it was revealed that there was no significant change for the non-BTX participants, *t*(11) = 0.638, *p* = 0.536, *d* = 0.385, but there was a significant drop in FSFI following treatment for BTX-treated frown-line only participants, *t*(10) = 3.012, *p* = 0.013, *d* = 1.905, and an near significant drop following treatment for BTX-treated frown-line-and-crow’s-feet participants, *t*(10) = 2.508, *p* = 0.067, *d* = 1.596. The full FSFI dataset (with all six subscales) is available for analysis at https://osf.io/qx4pa/.

## Discussion

Botulinum toxin is a powerful neurotoxin. Its effects on targeted muscle groups are effective for a wide range of medical conditions and it has rapidly become a popular cosmetic treatment product^1^. The current study demonstrates that the use of BTX for its cosmetic effects can have wide reaching psychological effects as well. The results inform us of these potential psychological effects but it also tells us more about the embodiment of emotions^18^.

The results reported here demonstrate that BTX treatment for glabellar frown lines leads to an improved mood in cosmetic patients, which supports for previous findings^5^. Unlike earlier research, the mood-improving effect of BTX was found here within participants (rather than between participants) using a baseline/post-treatment design. This confirms that the participants’ mood was changing after treatment rather than just happier people being more inclined to select BTX treatments. This is also supported by the RCT that have been carried out recently on its effectiveness as a treatment for depression^6–11^. The current finding is also consistent with the embodied-emotion based idea that interrupting the ability to frown reduces negative affect.

The current research explored mood further by looking at the effect that BTX treatment of crow’s feet has on mood. This treatment involves injections into the orbicularis oculi muscles and leads to a reduction in facial lines that are sometimes called laughter lines. Based on embodied emotion^18^, it was predicted that treating the laughter lines would lead to a lowering of a person’s mood. It happened that all of the participants who were BTX treated for crow’s feet were also BTX treated for frown lines. As such, in order to isolate any effect of treating crow’s feet, it makes sense to contrast those people who received BTX for frown lines only with those people who received the treatment for both frown lines and crow’s feet. The results of this comparison showed that the effect of having had a treatment for crow’s feet was to increase depression and anxiety scores relative to those who had only received the frown lines treatment.

The theory explaining why treating crow’s feet lowers mood is the same as that explaining why treatment of glabellar frown lines improves mood. Treatment of crow’s feet using BTX injections in the orbicularis oculi muscles leads to a lack of mobility in those muscles and the inability to smile using the eye muscles, that is, make a Duchenne smile. The strength of the facial feedback that a person would get when smiling would therefore be reduced relative to an untreated person and hence they feel less happy even when smiling. The current results show that the size of the negative effect of treating the crow’s feet appears to be similar in size to the positive effect of treating the frown lines and so for people having both treatments there is no net drop in mood. This finding demonstrates the potential for negative consequences of interfering with the embodiment of emotions. It is also an important consideration for anyone who is tempted to have only their laughter lines treated with BTX as this may lead to an increase in depression scores.

The second hypothesis tested was that a loss of mobility in the face as a result of BTX injections would be related to a loss in emotional-expression recognition ability, testing the concept that facial mimicry is important in emotional expression decoding. This effect had been shown previously but in an experiment that only assessed emotion recognition from the eye region^37^ and so it remained unclear as to whether whole face emotion recognition would be similarly affected. Two separate measures of emotion recognition were employed in the current quasi-experiment. The RMET confirmed the previous finding that emotion recognition from the eyes was affected by BTX injections. This research was extended by the finding that emotion recognition from the whole face was similarly affected by BTX injections as observed in the FER task.

The current findings from the FER and RMET are taken as evidence that the BTX treatment is interfering with the emotion-recognition pathways. The assumed pathway in question is the process of mimicking the expression being judged in order to better feel the emotion being expressed^31^. By preventing or reducing this mimicking process, the BTX treatment is reducing the accuracy with which facial expressions of emotion are recognised. The consequence of this effect is that people who have undergone BTX cosmetic treatment may be less able to interact effectively in social settings that might require interpretation of subtle facial cues.

Another question is whether people who have more BTX treatment show a greater detriment in emotional-expression recognition than those who have less BTX treatments. From the evidence collected here, both the FER test and the RMET show a larger detriment in emotional expression processing following treatment for crow’s feet and frown lines than for just frown lines. Care must be taken when interpreting this potential effect as it is possible it may be influenced by the different baseline abilities in the FER test. While the differences between the two BTX conditions in each task are non-significant, their combined effect is suggestive of the effect that people with frown lines and crow’s feet treated with BTX show a larger detriment in emotional-expression recognition than those who only have their frown lines treated. Fully evaluating this difference will require further research dedicated to answering this question, in particular assessing the total number of units of BTX received as well as the number of locations treated.

The final and most novel hypothesis that was tested concerned the expression of pleasure during sexual excitement. Previous research has shown that the muscle groups often targeted in cosmetic BTX treatments are the same ones that are associated with sexual excitement and orgasm^44^. Reduction of mobility of these muscles may therefore interfere with the expression and feedback of excitement during sexual activity. The current research provides support for this hypothesis in that participants reported that, following BTX treatment, there was a decrease in sexual function: in particular, orgasms were harder to achieve and were less satisfying.

The current study suggests that reducing the ability to make the facial expressions associated with sexual pleasure leads to a reduction in the reported feeling of pleasure associated with it. This finding demonstrates the importance of facial expressions during sexual intercourse. The results suggest that the facial expressions do not occur simply to communicate pleasure to a partner but they are an integral part of the feeling of pleasure and are important in the process of achieving orgasm. This demonstrates an important role for facial feedback within sexual intercourse and it is potentially a previously unimagined significant negative impact from cosmetic BTX treatments.

The current research only used self-report measures for analysing the effect of BTX treatments on sexual function. Given that the facial expressions during intercourse may have more than one role, future research should look at the wider effects that the treatment may have. In particular, it has been hypothesised that the female orgasm is part of pair-bonding^52^ and so communication of the orgasm through facial expressions will be an important aspect of emotional closeness. As such, future studies should look at the experience and satisfaction of the sexual partner of the person who has received the BTX treatment to investigate whether the loss of the communication element of the O face leads to a reduction in either their satisfaction or their emotional closeness.

A limitation of all parts of the current study is that the participants self-selected for the treatments that they received. This means that the findings do not reach the gold standard of a RCT. The justification for the current methodology is that it would not have been ethically acceptable to carry out this study as a RCT to test the current hypotheses because these propose that the BTX-treatments have negative effects, albeit in very specific domains. It would not be ethical to randomly give a proportion of the participants a treatment that may make them sad, make it difficult for them in social settings or reduce their sexual satisfaction. It is ethical, however, to study people as they do this to themselves anyway in their attempts to look younger. In spite of this limitation, the fact that the participants were tested before and after treatment and the treatments were compared with other similar treatments means that the research is superior to some earlier demonstrations of the psychological effects of BTX treatments.

A further limitation of the study was the lack of any male participants, which therefore limits any conclusions to a female population. There are no strong reasons why mood or emotion recognition might be differentially affected by BTX treatments in men than in women and so one might expect the effects to generalise to males but this remains unknown. As currently over 90% of cosmetic BTX treatments are administered to women^1^, it remains useful to gain an insight into how these treatments are affecting just this group. The findings regarding orgasm are particularly difficult to generalise from female to male as orgasms are different between the two sexes^52^. However, the corrugator muscle has been shown to be involved in the male orgasm as much as the female orgasm^44^ and so one would predict that the BTX-treatment effect would occur for males as well as females.

In conclusion, the current research used a prospective baseline/retest design with participants undergoing cosmetic treatments in order to assess the psychological effects of BTX treatments. The findings represent a significant addition to our knowledge concerning the impact of BTX cosmetic treatments. Equally, the findings make a significant contribution to the theory of embodied emotions demonstrating its importance in a previously untested domain of life. Specifically, it is demonstrated that targeted injections of BTX into facial muscles can affect a person’s mood in both positive and negative directions by treating either frown lines or laughter lines respectively. Further, a person’s social interactions can be negatively affected by these injections both generally, in terms of emotion recognition, and specifically in terms of sexual pleasure. Although this is a relatively small scale study, and further larger scale research is required, the sizes of these effects suggest that these powerful neurotoxins can have equally powerful psychological effects and consideration of these psychological effects is import for both practitioners and patients in the cosmetic treatment industry.

## Method

### Power calculation

Previous research has demonstrated that the effects of BTX, where present, can be large in size. An effect size of *d* = 1.410 has been found for depression^5^ whereas an effect size of *d* = 0.760 has been found for emotional expression recognition^37^. These estimates are based on between-participant analyses where no baseline was taken. The power of the current study was enhanced by using a within-participant design testing people before and after receiving cosmetic treatment. In this kind of design the effect size on depression has been shown to go up to *d* = 2.32^11^ and on emotion processing to be at *d* = 1.22^36^. Given this previous research, it was calculated that at least ten participants per group would provide sufficient power (α = 0.05, β = 0.80) to test the emotion recognition hypothesis and fewer still to test the depression hypothesis. A power calculation was not possible for the sexual function hypothesis and so this part of the research must be considered exploratory.

### Participants

Thirty six participants were recruited through social media, newspaper adverts, snowballing and local cosmetic clinics. All participants were female although the study was open to both males and females. These participants were planning on having some form of cosmetic treatment within the next month. This quasi-experiment was naturalistic in design with the participants selecting their own treatment. An exclusion criterion was having had a BTX cosmetic procedure within the last 12 months. Participants were tested once before their chosen cosmetic treatment and once at least a month after their treatment.

### Procedure

Participants took part in two identical testing sessions. The first of these sessions was at least 12 months since their last BTX cosmetic treatment and between 1 day and 1 month prior to undergoing a cosmetic treatment. The second of these sessions took place between 4 weeks and 8 weeks after treatment. The sessions began with a questionnaire about the cosmetic treatments that had been received or that they were planning to have. The remainder of the procedure consisted of a series of tests to assess mood, emotional expression recognition and sexual function.

The study was reviewed and approved for ethics by the Cardiff University, School of Psychology Research Ethics Committee and adhered to the ethical guidelines of the British Psychological Society. Informed consent was obtained from all participants. The data collected are available for inspection at https://osf.io/qx4pa/.

#### Mood questionnaire

Participants completed the Hospital Anxiety and Depression Scale (HADS)^51^. This consists of 14 questions with four alternative answers each. The answers of these questions were combined to provide a single value depicting the level of mood (higher values showing lower mood).

#### Reading the Mind in the Eyes test

This test was developed as a measure of emotion expression processing to be used with adults with autism^38^. Participants saw a series of 36 faces cropped such that only the eye region was visible. The participants’ task for each image was to select from a list of four possible emotions which emotion was the correct one being portrayed by the face. Participants could study the face for as long as they liked and the next trial began once a selection had been made. The task started with a practice item that was not included in the analysis.

#### Facial Emotion Recognition test

Six of the Ekman faces were used showing the emotions of happiness, sadness, surprise, fear, anger and disgust. These were morphed with the neutral version of the face to generate 4 levels of intensity of each expression: 100%, 75%, 50% and 25%^39^. The participants saw each of the 144 faces individually and responded as to which emotion was being presented (happiness, sadness, surprise, fear, anger, disgust or neutral). Participants could study each face for as long as they liked and the next trial began once a selection had been made.

#### Female Sexual Function Index

Participants completed the FSFI^50^. This consists of 19 questions about various aspects of sexual experience from desire to satisfaction. Answers were scored from 1 to 5 (lower numbers indicating lower sexual function) or they could record 0 if there had been no sexual activity. From these questions, an overall measure of sexual function was calculated.

### Design

The design was a baseline/retest evaluation of naturally observed cosmetic treatments. At retest, participants were categorised into one of three groups depending on the cosmetic treatment they had received between baseline and retesting (BTX frown lines only, BTX frown lines and crow’s feet or non-BTX). The main dependent variables were the changes in performance from baseline to retest on the questionnaires (HADS and FSFI) and emotion recognition tests (RMET and FER).
